# Use of 16S rRNA Gut Microbiome Analysis to Identify Shared Microbial Signatures Across Metabolic Syndrome Components in Obese Subjects

**DOI:** 10.3390/ijms27219701

**Published:** 2026-10-29

**Authors:** Lorenzo Antonetti, Susanna Longo, Marina Cardellini, Viviana Casagrande, Rossella Menghini, Ilaria Cicalini, Damiana Pieragostino, Eleonora Poggiogalle, Francesco Frigerio, Claudia Piciocchi, Vincenzo De Laurenzi, Silvia Savastano, Lorenzo Maria Donini, Geltrude Mingrone, Massimo Federici

**Affiliations:** 1 Department of Systems Medicine, University of Rome Tor Vergata, 00133 Rome, Italysusanna.longo@uniroma2.it (S.L.); cardellini@med.uniroma2.it (M.C.); casagrande@med.uniroma2.it (V.C.); menghini@med.uniroma2.it (R.M.); 2 Center for Advanced Studies and Technology (CAST), ‘G. D’Annunzio’ University of Chieti-Pescara, 66013 Chieti, Italy; 3 Department of Innovative Technologies in Medicine and Dentistry, ‘G. D’Annunzio’ University of Chieti-Pescara, 66013 Chieti, Italy; 4 Department of Experimental Medicine, Sapienza University, 00185 Rome, Italy; 5 Department for the Promotion of Human Sciences and Quality of Life, San Raffaele Roma University, Via di Val Cannuta 247, 00166 Rome, Italy; 6 Laboratory of Cardiovascular Endocrinology, IRCCS San Raffaele Roma, Via di Val Cannuta 247, 00166 Rome, Italy; 7 Unità di Endocrinologia, Diabetologia ed Andrologia, Dipartimento di Medicina Clinica e Chirurgia, Università degli Studi di Napoli Federico II, 80131 Naples, Italy; 8 Università Cattolica del Sacro Cuore, 00168 Rome, Italy; 9 Fondazione Policlinico Universitario A. Gemelli IRCCS, 00168 Rome, Italy; 10 Division of Diabetes & Nutritional Sciences, School of Cardiovascular and Metabolic Medicine & Sciences, King’s College London, London WC2R 2LS, UK; 11 IRCCS NeuroMED, 86077 Pozzilli, Italy

**Keywords:** microbiome, metabolic syndrome, obesity, insulin resistance

## Abstract

Gut microbiota (GM) alterations and related metabolic outputs have been implicated in metabolic syndrome (MetS) and early cardiometabolic risk. However, the associations between GM alterations and MetS, and their relationship with the marked interindividual variability in cardiovascular risk, remain incompletely defined. We conducted a cross-sectional study in individuals with obesity (n = 176) to evaluate associations between GM features and MetS clinical traits using 16S amplicon profiling data. Prevalence of individual MetS traits was 37.4% for hyperglycemia, 23.4% for hypertriglyceridemia, 54.0% for hypertension, and 52.1% for low HDL cholesterol, with 51.1% of participants meeting criteria for MetS overall. Comparative analysis of 16S rRNA amplicon sequencing data identified a shared gut microbial signature comprising 45 ASVs across MetS clinical components. Members of *Prevotellaceae* and *Ruminococcaceae* were consistently reduced in MetS, whereas *Veillonella* and *Streptococcus* were enriched. Reduced microbial alpha diversity was observed in individuals with MetS. PICRUSt2-based functional inference indicated lower representation of pathways related to complex carbohydrate utilization and short-chain fatty acid (SCFA) metabolism in MetS-positive individuals. Our results, derived from two independent datasets, indicate that MetS is associated with a depletion of bacterial taxa involved in complex carbohydrate metabolism and SCFA production.

## 1. Introduction

Metabolic syndrome (MetS) is defined by the co-occurrence of interrelated metabolic abnormalities, including central adiposity, insulin resistance, dyslipidemia, and elevated blood pressure, which together reflect a state of systemic metabolic dysfunction and confer a substantially increased risk of cardiovascular disease and adverse metabolic outcomes, such as type 2 diabetes and cardiometabolic complications [1,2]. However, individuals fulfilling the same diagnostic criteria may exhibit substantial heterogeneity in cardiometabolic risk, indicating that MetS encompasses diverse underlying biological profiles [1,3].

The global prevalence of MetS has risen substantially over recent decades, reflecting epidemiological trends characteristic of modern societies and paralleling widespread lifestyle changes, including shifts in environmental exposures, dietary patterns, and daily behaviors that collectively promote metabolic dysregulation [4]. Global analyses have reported a marked increase in the burden of metabolic diseases between 2000 and 2019, with substantial growth in both prevalence and attributable disability and mortality worldwide [5].

Recent advances have highlighted biological systems that may mediate the effects of lifestyle on metabolic health, with the gut microbiota (GM) emerging as a key interface between environmental exposures and host metabolism [6,7]. Experimental and population-based studies have shown that GM-associated shifts accompany early metabolic alterations, including insulin resistance and low-grade inflammation, suggesting a functional involvement of the microbiota in cardiometabolic risk. Importantly, these microbiota-associated alterations are detectable during subclinical stages of metabolic dysfunction, before the development of overt metabolic syndrome and type 2 diabetes, supporting a role of GM-related features in early cardiometabolic risk stratification [8].

Alterations in GM composition and function have been linked to metabolic disease through disruption of host–microbiota interactions and changes in the production of microbially derived metabolites, which represent intermediates or end products of bacterial metabolism originating from dietary or host-derived substrates [8,9].

These metabolites, including short-chain fatty acids, bile acid derivatives, and amino acid-derived compounds, influence host metabolic pathways related to glucose handling, lipid metabolism, and inflammatory regulation, thereby providing a mechanistic link between environmental exposures and metabolic dysfunction [10,11,12]. Increasing evidence indicates that functional changes in microbial metabolic capacity may be more closely related to metabolic phenotypes than taxonomic shifts alone, highlighting the relevance of integrative approaches that combine microbiota profiling with metabolic readouts and methods capable of quantifying or estimating the functional contribution of microbial communities [6,13].

In this context, the GM and its metabolic outputs have been proposed as candidate early biomarkers of cardiometabolic risk and as potential targets for preventive strategies [8,14]. However, the mechanistic links through which GM alterations influence MetS remain incompletely defined. Moreover, the relationship between microbial features and MetS, considered both as an overall condition and at the level of its individual clinical traits, has not been systematically characterized with respect to early cardiometabolic risk. Individual components of MetS, including hyperglycemia, dyslipidemia, and elevated blood pressure, reflect partially distinct pathophysiological processes and may therefore exhibit differential associations with gut microbial features [3,15].

Accordingly, this cross-sectional study evaluated associations between gut microbiome features and metabolic syndrome (MetS), considered both as an overall condition and through its individual clinical components. We integrated 16S rRNA gene amplicon sequencing approaches that are currently used, or increasingly being adopted, in clinical research and practice [16] to explore the potential translational relevance of MetS-associated microbial features.

## 2. Results

### 2.1. Clinical Characteristics

All participants included in the study exhibited abdominal obesity (100%). The proportion of individuals positive for the other four MetS traits was 37.4% for hyperglycemia, 23.4% for hypertriglyceridemia, 54.0% for hypertension, and 52.1% for low HDL cholesterol (Table 1). Overall, 51.1% of the participants were positive for MetS.

### 2.2. 16S Gene Amplicon Sequencing

**Figure 1:**
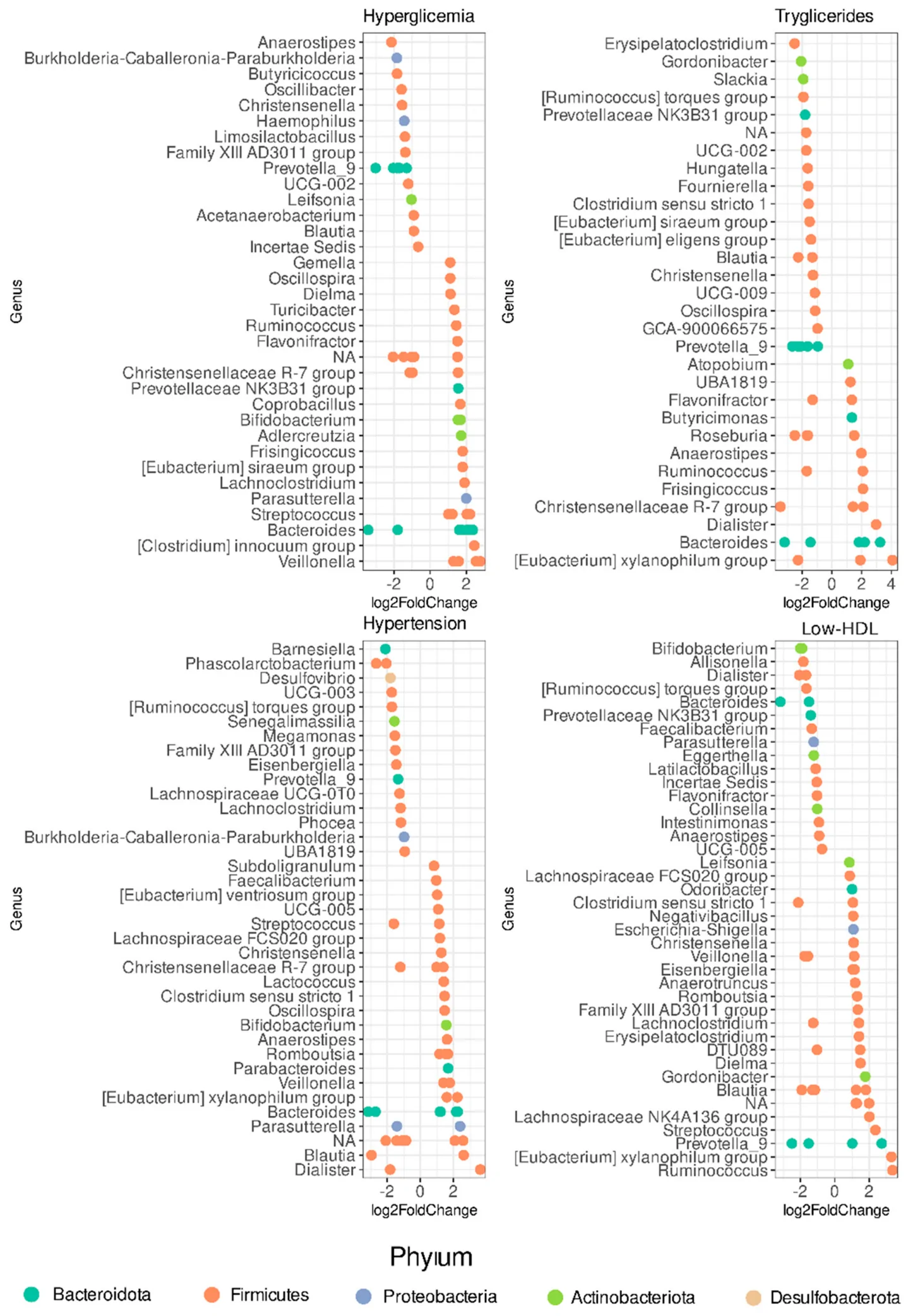
Comparative analysis for each MetS clinical trait revealed several ASVs significantly differentially abundant in each comparison. Point colors indicate the corresponding phylum, and the direction of log2FC reflects relative abundance in trait-positive versus trait-negative individuals.

To investigate gut microbiota differences between individuals positive for a specific MetS trait and those without, 16S amplicon profiling of fecal samples was performed. Differential abundance analysis using DESeq2 revealed numerous significantly altered ASVs, and in each comparison, several taxa belonging to the phylum Firmicutes were both increased and decreased (Figure 1 and Figure S1).

**Figure 2:**
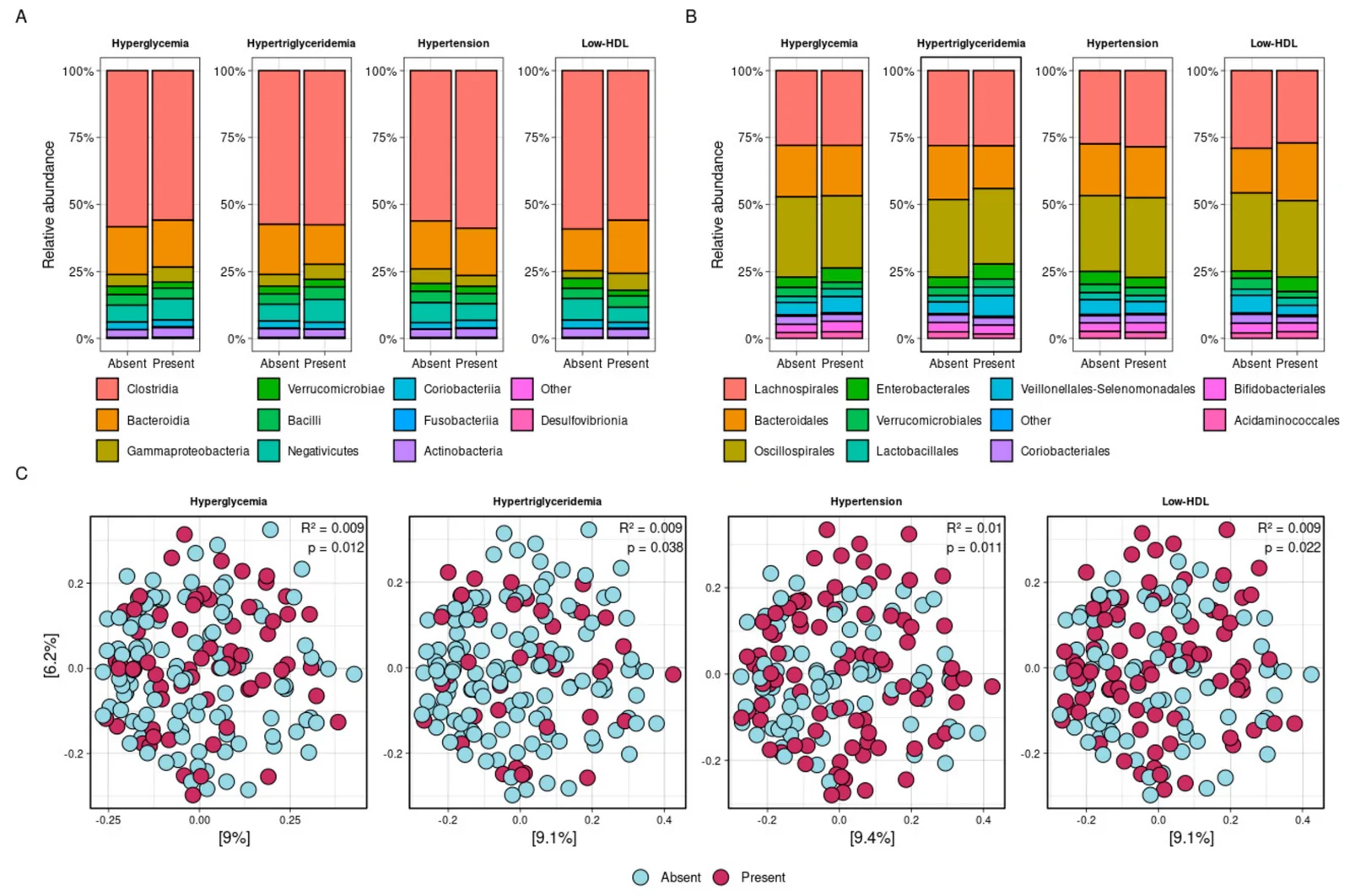
(**A**,**B**) Class and order-level composition of the gut microbiota across metabolic traits. Stacked bar plots display the relative abundance (%) of the 10 most prevalent taxa classified based on the absence of each metabolic syndrome (MetS) component. (**C**) Beta-diversity of gut microbiota according to metabolic traits. Principal Coordinates Analysis (PCoA) based on Bray–Curtis dissimilarities was performed to evaluate differences in microbial community composition between groups. Each point represents an individual sample. Percentages on the axes indicate the proportion of variance explained by each coordinate. Statistical significance of compositional differences between groups was assessed using PERMANOVA (adonis2, 999 permutations). Reported R^2^ values indicate the proportion of community variance explained by the grouping variable, and *p*-values denote the significance of these differences.

Within Firmicutes, the most represented and variable class was *Clostridia*, particularly the orders *Lachnospirales* and *Oscillospirales*; among Bacteroidota, most changes involved *Bacteroidales* (Figure 1 and Figure 2A,B). Bray–Curtis PCoA with PERMANOVA demonstrated statistically significant (*p* < 0.05) separation in GM composition between individuals with and without each metabolic trait (Figure 2C). PERMANOVA R^2^, exact *p*-values, and Benjamini–Hochberg-adjusted *p*-values are reported in Supplementary Table S1. Homogeneity of multivariate dispersion was confirmed for all traits. Alpha diversity, assessed using observed features, ACE, and Fisher’s alpha, was significantly higher in subjects without MetS (*p* < 0.05; Figure S2). For the other metabolic traits, alpha diversity showed a non-significant increasing trend in metabolically healthier individuals.

**Figure 3:**
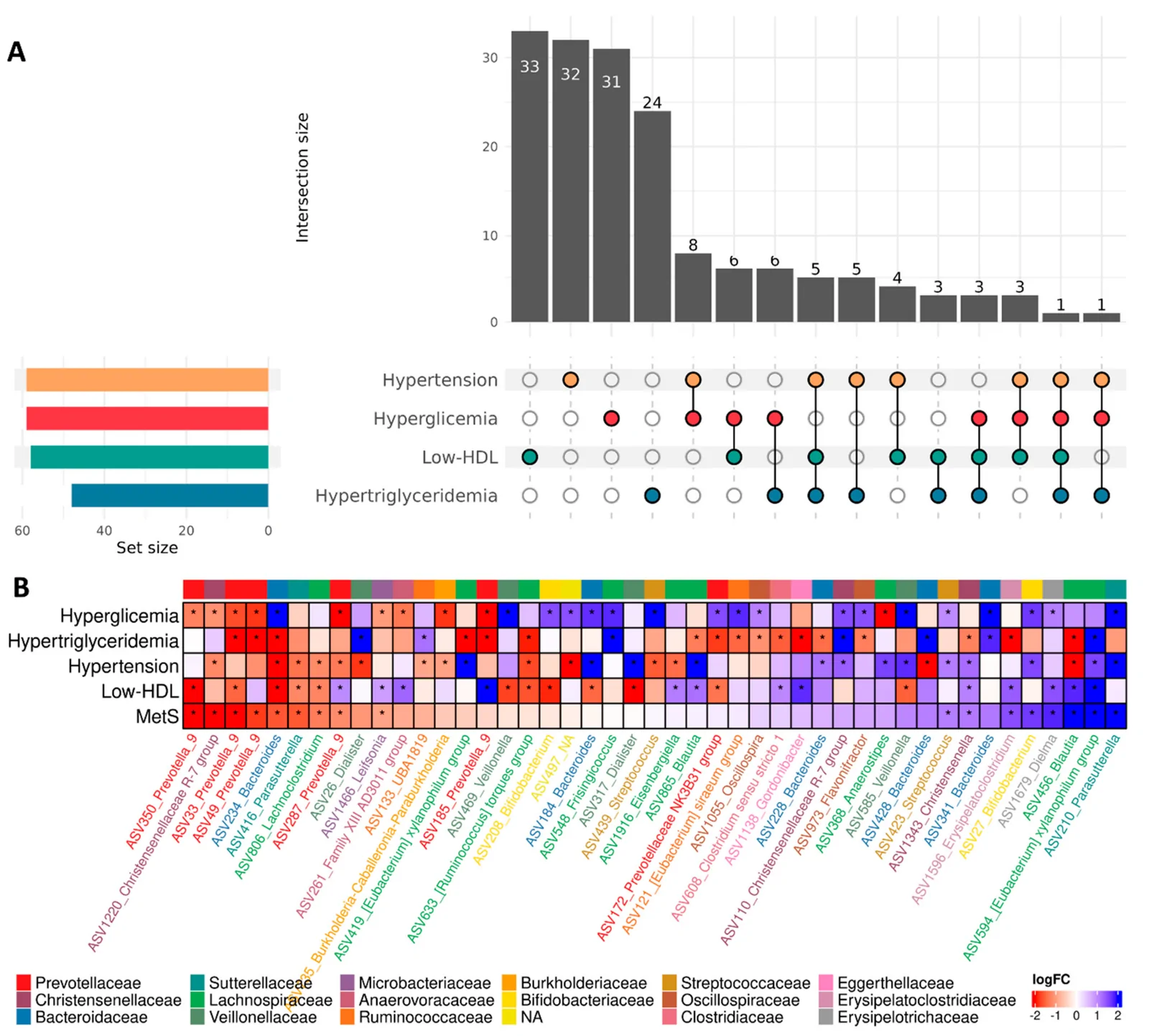
(**A**) Upset plot showing ASVs shared with single or multiple components of MetS. Horizontal bars indicate the total number of ASVs associated with each MetS component. Vertical bars represent the number of ASVs shared between one or multiple components. Connected dots indicate which specific traits share a given group of ASVs. (**B**) Heatmap showing ASVs shared across at least 2 different MetS components. Each row represents an individual ASV, and each column corresponds to a specific MetS trait. Colors indicate the direction and magnitude of log2 fold change for each ASV within a given trait, with blue shades indicating an increase and red shades indicating a decrease in cases compared with controls. * denotes statistical significance at adjusted *p*-value < 0.05.

Comparison of the sets of significantly associated ASVs revealed that numerous taxa were associated with either individual or multiple MetS components. In particular, we identified 33, 32, 31, and 24 ASVs associated only with low-HDL cholesterol, hypertension, hyperglycemia and hypertriglyceridemia, respectively (Figure 3A). A total of 45 ASVs were found to be shared across at least two MetS traits (Figure 3B). Their log_2_ fold-change profiles highlighted heterogeneous behavior across components, with ASVs assigned to *Dialister*, *Christenesellaceae R7-group*, *Bacteroides*, *Parasutterella* and *Bifidobacterium* showing recurrent trait-specific variation. Several members of *Prevotella_9*, *Ruminococcaceae UBA1819* and *Ruminococcus torques group* tended to decrease across MetS components, whereas ASVs belonging to *Veillonella*, *Erysipelatoclostridium*, [*Eubacterium*] *xylanophilum group*, *Anaerostipes* and *Streptococcus* were frequently increased. Additional shifts involved taxa affiliated with *Parabacteroides*, *Dialister*, *Roseburia* and *Clostridium_sensu_stricto1*.

**Figure 4:**
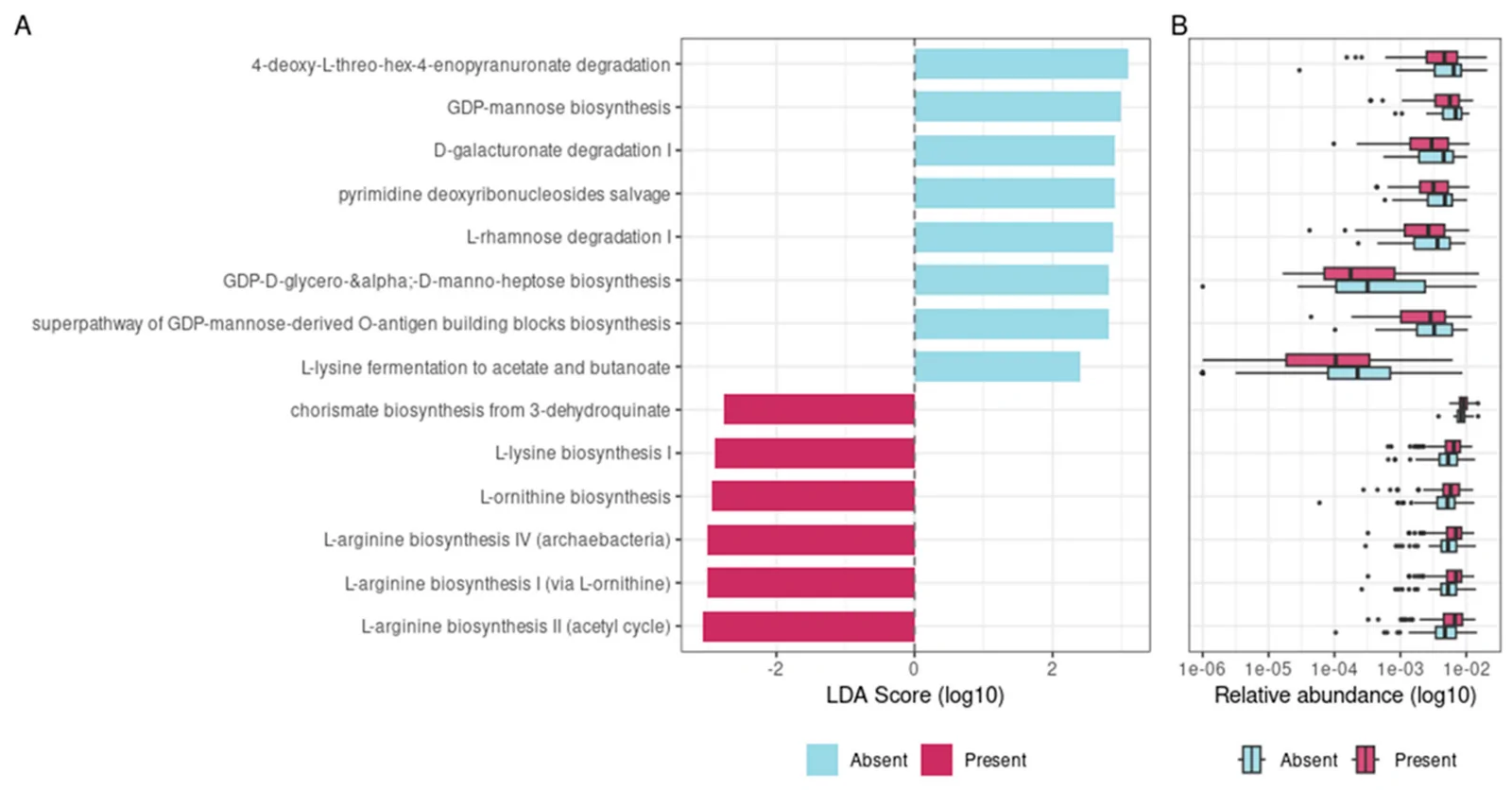
PICRUSt2-predicted functional pathway enrichment in subjects with and without metabolic syndrome (MetS). (**A**) Linear Discriminant Analysis (LDA) scores for MetaCyc pathways significantly associated with MetS status, as identified by LEfSe. Bars extending to the right indicate enrichment in subjects without MetS (absent, light blue); bars extending to the left indicate enrichment in subjects with MetS (present, dark red). Pathways are ordered by signed LDA score. (**B**) Relative abundances (log_10_ scale) of the significant pathways stratified by MetS status.

PICRUSt2 functional profiling identified several inferred microbial pathways differing between MetS-positive and MetS-negative individuals (Figure 4A,B). In particular, metabolically healthy subjects showed a significant enrichment of predicted functional potential involved in pectin degradation pathways, including L-rhamnose, D-galacturonate, and 4-deoxy-L-threo-enopyranuronate degradation, and in acetate production.

### 2.3. External Validation

**Figure 5:**
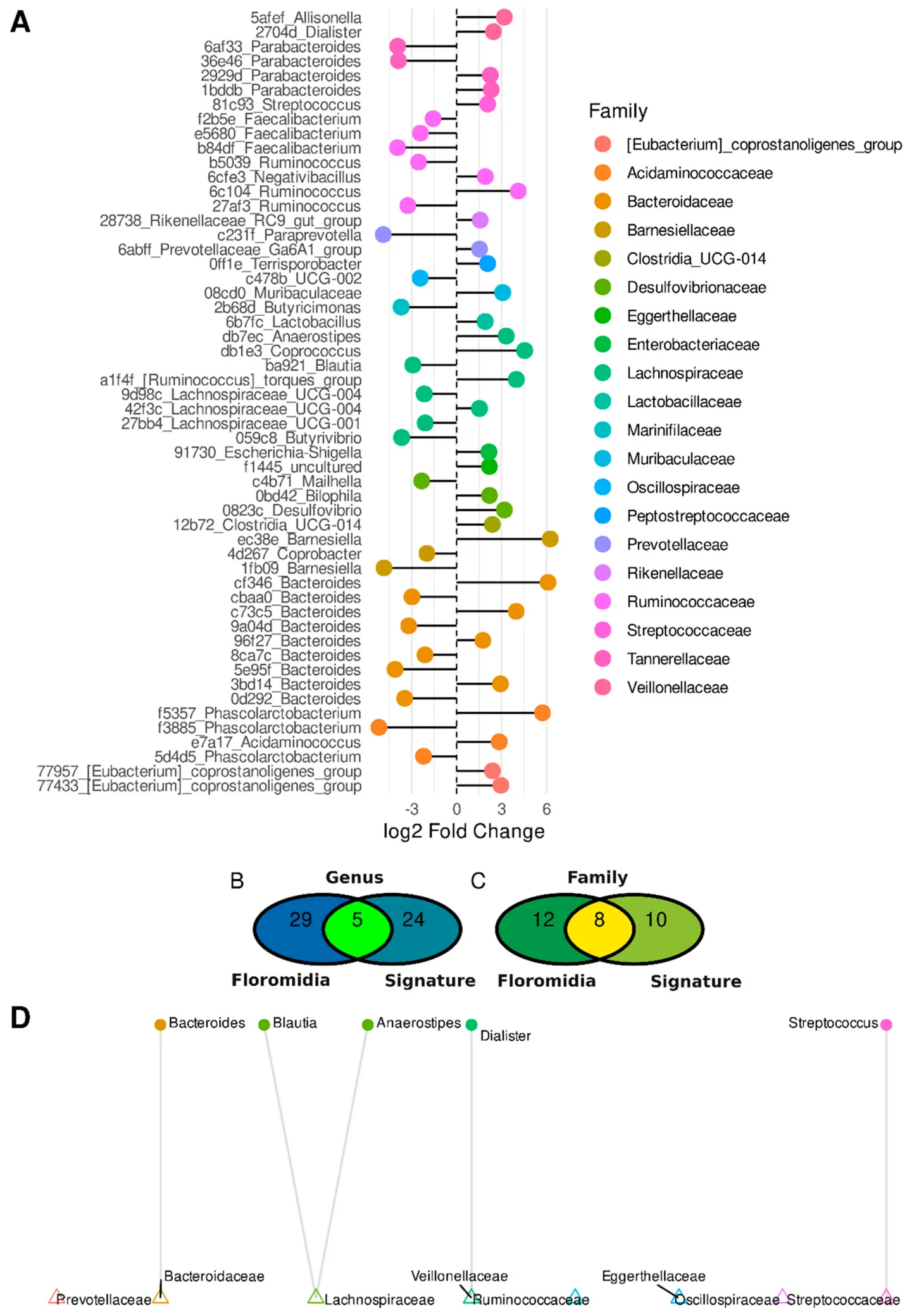
(**A**) Differential abundance results comparing MetS-positive and MetS-negative subjects from the Floromidia dataset. Points represent individual microbial taxa, positioned according to their log2 fold change; positive values indicate higher abundance in MetS-positive individuals, while negative values indicate higher abundance in MetS-negative individuals. Point colors denote the corresponding taxonomic family. Only taxa with adjusted *p*-value < 0.05 are shown. (**B**,**C**) Venn diagrams illustrating the overlap in microbial taxa detected in the validation set and those identified in the signature. Panel (**B**) reports the distribution of shared and unique genera across datasets, whereas Panel (**C**) shows the corresponding overlap at the family level. Values indicate the number and proportion of taxa within each set and in their intersection. (**D**) Bipartite network representation of taxa common to both datasets. Triangles denote bacterial families and circles represent genera; edges connect each family to its constituent genera. Node colors reflect taxonomic family assignment.

To validate our findings, we stratified participants in the external validation cohort according to MetS status. Differential abundance analysis identified 55 significant taxa (Figure 5A). Multiple ASVs belonging to the *Lachnospiraceae_UCG_004* and *Lachnospiraceae_UCG_001*, *Faecalibacterium*, *Butyrivibrio*, *Butyricimonas*, *Blautia* and *Ruminococcus* genera were significantly reduced in MetS-positive subjects. In contrast, ASVs assigned to the genera *Streptococcus*, *Escherichia-Shigella*, [*Eubacterium*]*_coprostanoligenes_group*, and *Acidaminococcus* were increased in MetS-positive subjects. Members of the genera *Bacteroides* and *Parabacteroides* were distributed on both sides of the log2 fold-change axis, indicating heterogeneous responses within these taxa. Comparison with the external validation cohort showed that five genera (Figure 5B) and eight families (Figure 5C) were shared between the validation set and the signature (Figure 3B). In Figure 5D, the shared genera, *Bacteroides*, *Blautia*, *Anaerostipes*, *Dialister*, and *Streptococcus*, are shown together with their respective families. These correspond to *Prevotellaceae*, *Bacteroidaceae*, *Lachnospiraceae*, *Ruminococcaceae*, *Eggerthellaceae*, *Veillonellaceae*, *Oscillospiraceae*, and *Streptococcaceae*.

## 3. Discussion

The marked interindividual variability in cardiometabolic risk among individuals with MetS has increased interest in gut microbiota (GM) alterations as potential markers associated with metabolic dysfunction. In this cross-sectional study of obese individuals, we evaluated GM features in relation to MetS and its individual clinical traits.

GM remodeling in metabolic syndrome has been attributed to environmental and host influences, including habitual diet and low-grade systemic inflammation, which alter substrate availability and gut ecosystem dynamics, promote dysbiosis, and disrupt intestinal barrier integrity, thereby facilitating microbial metabolite translocation and inflammatory signaling linked to cardiometabolic risk [6,17,18]. Gut microbiota-derived metabolites contribute to immune and intestinal barrier homeostasis, whereas dysbiosis may impair barrier integrity and promote immune dysregulation and systemic exposure to microbial products, thereby sustaining the low-grade inflammation associated with MetS [19]. Comparative 16S amplicon sequencing analyses between individuals positive and negative for specific MetS traits, as well as for MetS overall, identified numerous ASVs belonging to multiple bacterial genera that were significantly associated with metabolic status, consistent with the observed beta-diversity separation between groups.

We identified a MetS-associated microbial signature through intersection analysis, comprising 45 ASVs shared across at least two MetS traits. Members of *Prevotella_9* and *Ruminococcaceae* (including *UBA1819* and the *Ruminococcus torques group*) consistently decreased across MetS components, while ASVs assigned to genera such as *Veillonella* and *Streptococcus* were frequently increased in MetS-positive subjects. Members of *Prevotellaceae* and *Ruminococcaceae* are well recognized for their role in the fermentation of microbiota-accessible carbohydrates (MACs) and short-chain fatty acid (SCFA) production [20,21] and have been negatively associated with MetS [15], whereas *Veillonella* and *Streptococcus* have been positively associated with MetS clinical components [12,15]. Additional shifts involved taxa affiliated with *Parabacteroides*, *Dialister*, *Roseburia* and *Clostridium_sensu_stricto1*, *Erysipelatoclostridium*, [*Eubacterium*] *xylanophilum group* and *Anaerostipes*. Notably, the reduction in *Ruminococcaceae* and the enrichment of *Streptococcus* were also independently observed in the external validation dataset as well as a modulation of members of *Prevotellaceae*, *Bacteroidaceae* (*Bacteroides*), *Lachnospiraceae* (*Blautia*, *Anaerostipes*), *Veillonellaceae* (*Dialister*), *Eggerthellaceae* and *Oscillospiraceae*. Given the differences in sequencing platforms between the two cohorts, concordance was interpreted primarily at the genus level, with family-level comparisons used to assess broader and potentially more robust taxonomic patterns across datasets.

Microbial richness is generally considered an integrative indicator of gut ecosystem stability and host metabolic health, and reduced microbial alpha diversity has been consistently reported in MetS [15,17]. We observed that alpha-diversity indices (Observed features, ACE, and Fisher’s index) were significantly reduced in individuals with MetS overall, while showing a non-significant decreasing trend across the remaining metabolic traits.

Dietary microbiota-accessible carbohydrates (MACs) and their microbial fermentation into short-chain fatty acids (SCFA) are involved in metabolic homeostasis and have been reported to be altered in MetS [12,22]. Accordingly, functional prediction analysis focused on the 45 ASVs shared across multiple MetS traits indicated that MetS-positive individuals had a lower inferred abundance of pathways involved in microbiota-accessible carbohydrate (MAC) utilization, including pectin degradation pathways such as L-rhamnose, D-galacturonate, and 4-deoxy-L-threo-enopyranuronate degradation, together with a reduced inferred potential for SCFA-related metabolism. Although our analyses were adjusted for age, sex, and batch effects, residual confounding by unmeasured or incompletely characterized clinical and lifestyle factors, including medication use, dietary habits, smoking, probiotic or antibiotic exposure, and gastrointestinal conditions, cannot be excluded and should be considered when interpreting the observed microbiome–MetS associations. Microbiome-modulating interventions, including dietary, prebiotic, and probiotic approaches, have shown potential effects on metabolic outcomes, although current evidence remains heterogeneous and the therapeutic significance of the specific taxa identified here requires further investigation [23]. In addition, the study population consisted exclusively of individuals with obesity, which limits the generalizability of the findings beyond this population. The taxonomic resolution of 16S rRNA gene sequencing is also limited, particularly at lower taxonomic levels, and the relatively small size of the external validation cohort (n = 37) should be taken into account when interpreting the reproducibility of the identified microbial signatures. Furthermore, PICRUSt2-based functional profiling provides an inference of microbial functional potential from 16S rRNA gene sequencing data and does not represent a direct measurement of microbial functional activity.

In conclusion, in individuals with obesity, our results, derived from two independent datasets, suggest that MetS is associated with a depletion of bacterial taxa potentially involved in complex carbohydrate metabolism and SCFA production.

## 4. Methods

### 4.1. Human Cohort

MICROBESOMICS is an exploratory study to investigate OMICS signatures. All subjects gave written informed consent, validated and approved by the ethical committee of Policlinico Tor Vergata University of Rome (Comitato Etico Indipendente, approval number RS 74.20). The study cohort comprised 176 individuals with obesity. Exclusion criteria included prior bariatric surgery, inflammatory bowel disease, probiotic use, recent antibiotic use, and intestinal cancer.

### 4.2. Outcomes

MetS was defined in men and women according to the American Heart Association/National Heart Lung and Blood Institute modified NCEP-ATP-III guidelines [24] requiring the presence of ≥3 of the following: waist circumference > 102 cm in men or 88 cm in women; triglyceride levels ≥ 150 mg/dL or treatment for dyslipidemia; HDL levels < 40 mg/dL in men or <50 mg/dL in women or treatment for dyslipidemia; fasting plasma glucose levels ≥ 100 mg/dL or treatment for diabetes; and blood pressure levels ≥ 130/85 mmHg or treatment for hypertension. Practically, these guidelines differ from the NCEP-ATP-III guidelines for the elevated fasting glucose criterion; the threshold is reduced from 110 to 100 mg/dL, which corresponds to the recently modified American Diabetes Association criteria for impaired fasting glucose. MetS controls had ≤2 components. Only individuals with non-missing data for the majority of components (three of five) were included as MetS cases or controls in analyses; for example, an individual having three of five components but missing data for the other two was included as a MetS case, and an individual not meeting criteria for three of the five components, even if the other two were missing, was included as a control. For analysis involving MetS components, binary variables were created using the above criteria to reflect either the presence or absence of the condition in individuals with non-missing data for that component.

### 4.3. 16S rRNA Gene Amplicon Sequencing

Libraries were prepared by following Illumina 16S Metagenomic Sequencing Library Preparation protocol in two amplification steps: an initial PCR amplification using locus-specific PCR primers and a subsequent amplification that integrates relevant flow-cell binding domains and unique indices (NexteraXT Index Kit, Illumina, San Diego, CA, USA, FC-131-1001/FC-131-1002). Primer sequences used were as follows:

16S-341F 5′-CCTACGGGNBGCASCAG-3′;

16S-805R 5′-GACTACNVGGGTATCTAATCC-3′.

Libraries were sequenced on NovaSeq instrument (Illumina, San Diego, CA, USA) using 250-bp paired-end mode. Only the pairs of reads containing both primers were selected with cutadapt = 4.1 with additional parameters—overlap of 10, times 2, error-rate of 0.15, trim-n, and max-n of 0. Amplicon Sequence Variants (ASV) analysis was performed using the following packages in R version 4.1.3 (10 March 2022):

- dada2 v. 1.22.0 [25];
- phyloseq v. 1.38.0 [26].

16S classification up to the level of Genus was obtained using Silva v138 database and the Bayesian classifier of Ribosomal Database Project (RDP Naïve Bayesian Classifier for Rapid Assignment of rRNA Sequences into the New Bacterial Taxonomy) available in the DADA2 package (kmer size 8, bootstrapping Iteration: 100, bootstrapping confidence: 50). Taxonomic classification was attributed by exact match between ASVs and a database of sequences which defines known genus-species associations for such sequences. Low-abundance and low-variance ASVs were excluded prior to downstream analyses, resulting in a final dataset of 564 ASVs. The resulting count matrix was subsequently processed in R using the phyloseq package.

#### 4.3.1. Differential Analysis of ASV Count

ASV counts were analyzed using DESeq2 after conversion through the phyloseq package and variance stabilizing normalization [27,28]. Differential abundance analyses were performed using Wald tests in models adjusted for age, sex, and batch effects. *p*-values were corrected for multiple testing using the Benjamini–Hochberg method.

#### 4.3.2. Alpha and Beta Diversity

Alpha-diversity was quantified using observed richness, ACE, and Fisher indices with vegan R package using rarefied count data [29]. For Beta diversity, microbial count data were transformed into Bray–Curtis dissimilarity matrices and visualized using Principal Coordinates Analysis. Statistical significance of community-composition differences between groups (trait present vs. absent) was tested using PERMANOVA (adonis2 function, 999 permutations). *p*-values across the five metabolic trait comparisons were corrected using the Benjamini–Hochberg method. Homogeneity of multivariate dispersion was assessed for each trait using betadisper followed by permutation testing.

#### 4.3.3. Metagenome Functions Prediction

Functional pathway prediction was performed from 16S rRNA gene sequencing data using PICRUSt2 (v2.5.1) [30]. Differential pathway enrichment analyses were subsequently conducted using LEfSe 1.1.2 with standard parameters.

### 4.4. Validation Cohort

The FLOROMIDIA database was already described [31,32]. The human subject cohort comprised 37 subjects recruited at the Department of Medicine, Policlinico Tor Vergata. MetS was defined using the same diagnostic criteria applied in the discovery cohort. Basic demographic and metabolic characteristics of the validation cohort are reported in Supplementary Table S2.

Briefly, stool samples were processed for 16S rDNA V3–V4 sequencing (MiSeq 2 × 300 bp) following standard dual-PCR library preparation. Reads were denoised with DADA2 and taxonomically assigned using a SILVA-trained Naïve Bayes classifier in QIIME2. Downstream processing was performed in R 4.6.1 and MicrobiomeAnalyst 2.0.

### 4.5. Statistical Analysis

Continuous variables are reported as mean ± standard deviation or median values according to data distribution, whereas categorical variables are presented as percentages. Normality was assessed using the Shapiro–Wilk test. Between-group comparisons were performed using one-way ANOVA or Kruskal–Wallis tests for continuous variables and χ^2^ tests for categorical variables. Multiple testing correction was performed using the Benjamini–Hochberg method, with adjusted *p*-values ≤ 0.05 considered statistically significant.

## Figures and Tables

**Table 1:** Baseline characteristics of the cohort. Data are presented as mean and standard deviation or as percentages. Sample size varies across variables because of missing data; the number of available observations is reported for each variable. BMI: body mass index; SBP: systolic blood pressure; DBP: diastolic blood pressure; FPG: fasting plasma glucose; HbA1c: glycated hemoglobin; HDL: high-density lipoprotein; LDL: low-density lipoprotein; HOMA: homeostatic model assessment; QUICKI: quantitative insulin sensitivity check index; MetS: metabolic syndrome. Sex: percentage of female participants. SCORE2 was calculated only for participants aged 40–69 years for whom all variables required for score calculation were available.

	N	Mean	Sd
Age (year)	176	50.36	10.82
BMI	176	38.23	5.72
Waist (cm)	170	117.31	16.16
Hip (cm)	118	120.96	13.70
SBP (mmHg)	163	126.21	13.98
DBP (mmHg)	163	79.12	10.59
FPG (mg/dL)	160	97.63	17.60
Score2	127	4.49	2.84
FIB4	169	0.85	0.44
HOMA	153	5.62	4.52
QUICKI	148	0.31	0.04
Matsuda	133	2.70	2.02
HbA1c (mmol/mol)	159	38.62	6.33
HDL (mg/dL)	167	49.25	12.80
LDL (mg/dL)	165	122.37	36.75
Triglycerides (mg/dL)	167	135.49	80.16
Sex (%)	176	67.61	
Hyperglycemia (%)	174	37.36	
Hypertriglyceridemia (%)	167	23.35	
Hypertension (%)	161	54.04	
Abdominal obesity (%)	176	100.00	
Low-HDL (%)	167	52.10	
MetS (%)	176	51.14	

## Data Availability

The datasets generated and/or analysed during the current study are available from the corresponding author upon reasonable request and can be accessed at http://bit.ly/4tYexxS (accessed on 10 January 2026).

## Supplementary Materials

The following supporting information can be downloaded at: https://www.mdpi.com/article/10.3390/ijms27219701/s1.

## References

[1] Neeland I.J., Lim S., Tchernof A., Gastaldelli A., Rangaswami J., Ndumele C.E., Powell-Wiley T.M., Després J.P. (2024). Metabolic Syndrome. Nat. Rev. Dis. Primers.

[2] Eckel R.H., Grundy S.M., Zimmet P.Z. (2005). The Metabolic Syndrome. Lancet.

[3] Lonardo A. (2023). The Heterogeneity of Metabolic Syndrome Presentation and Challenges This Causes in Its Pharmacological Management: A Narrative Review Focusing on Principal Risk Modifiers. Expert Rev. Clin. Pharmacol..

[4] Noubiap J.J., Nansseu J.R., Nyaga U.F., Ndoadoumgue A.L., Ngouo A.T., Tounouga D.N., Tianyi F.L., Foka A.J., Lontchi-Yimagou E., Nkeck J.R. (2026). Worldwide Trends in Metabolic Syndrome from 2000 to 2023: A Systematic Review and Modelling Analysis. Nat. Commun..

[5] Chew N.W.S., Ng C.H., Tan D.J.H., Kong G., Lin C., Chin Y.H., Lim W.H., Huang D.Q., Quek J., Fu C.E. (2023). The Global Burden of Metabolic Disease: Data from 2000 to 2019. Cell Metab..

[6] Dabke K., Hendrick G., Devkota S. (2019). The Gut Microbiome and Metabolic Syndrome. J. Clin. Investig..

[7] Wutthi-in M., Cheevadhanarak S., Yasom S., Kerdphoo S., Thiennimitr P., Phrommintikul A., Chattipakorn N., Kittichotirat W., Chattipakorn S. (2020). Gut Microbiota Profiles of Treated Metabolic Syndrome Patients and Their Relationship with Metabolic Health. Sci. Rep..

[8] Gabriel C.L., Ferguson J.F. (2023). Gut Microbiota and Microbial Metabolism in Early Risk of Cardiometabolic Disease. Circ. Res..

[9] Olalekan S.O., Bakare O.O., Osonuga I.O., Faponle A.S., Adegbesan B.O., Ezima E.N. (2024). Gut Microbiota-Derived Metabolites: Implications for Metabolic Syndrome and Therapeutic Interventions. Egypt. J. Intern. Med..

[10] Cai J., Rimal B., Jiang C., Chiang J.Y.L., Patterson A.D. (2022). Bile Acid Metabolism and Signaling, the Microbiota, and Metabolic Disease. Pharmacol. Ther..

[11] Zhu X., Dai X., Zhao L., Li J., Zhu Y., He W., Guan X., Wu T., Liu L., Song H. (2024). Quercetin Activates Energy Expenditure to Combat Metabolic Syndrome through Modulating Gut Microbiota-Bile Acids Crosstalk in Mice. Gut Microbes.

[12] Karlsson F., Tremaroli V., Nielsen J., Bäckhed F. (2013). Assessing the Human Gut Microbiota in Metabolic Diseases. Diabetes.

[13] Boicean A., Ichim C., Sasu S.M., Todor S.B. (2025). Key Insights into Gut Alterations in Metabolic Syndrome. J. Clin. Med..

[14] Kappel B.A., Federici M. (2019). Gut Microbiome and Cardiometabolic Risk. Rev. Endocr. Metab. Disord..

[15] He Y., Wu W., Wu S., Zheng H.M., Li P., Sheng H.F., Chen M.X., Chen Z.H., Ji G.Y., Zheng Z.D.X. (2018). Linking Gut Microbiota, Metabolic Syndrome and Economic Status Based on a Population-Level Analysis. Microbiome.

[16] Tegegne H.A., Savidge T.C. (2025). Gut Microbiome Metagenomics in Clinical Practice: Bridging the Gap between Research and Precision Medicine. Gut Microbes.

[17] Qin Q., Yan S., Yang Y., Chen J., Li T., Gao X., Yan H., Wang Y., Wang J., Wang S. (2021). A Metagenome-Wide Association Study of the Gut Microbiome and Metabolic Syndrome. Front. Microbiol..

[18] Araujo R., Borges-Canha M., Pimentel-Nunes P. (2022). Microbiota Modulation in Patients with Metabolic Syndrome. Nutrients.

[19] Dogra K.A., Sharma M., Ghosh N., Kaur G., Singh J., Sinha P. (2026). Gut Microbiota and Immune Modulation: Role in Neurodegenerative Disorders and Cancer. Mol. Biol. Rep..

[20] Maier T.V., Lucio M., Lee L.H., Verberkmoes N.C., Brislawn C.J., Bernhardt J., Lamendella R., McDermott J.E., Bergeron N., Heinzmann S.S. (2017). Impact of Dietary Resistant Starch on the Human Gut Microbiome, Metaproteome, and Metabolome. mBio.

[21] Kim Y.J., Jung D.H., Park C.S. (2024). Important Roles of *Ruminococcaceae* in the Human Intestine for Resistant Starch Utilization. Food Sci. Biotechnol..

[22] de Groot P.F., Frissen M.N., de Clercq N.C., Nieuwdorp M. (2017). Fecal Microbiota Transplantation in Metabolic Syndrome: History, Present and Future. Gut Microbes.

[23] Singh J., Pooja, Kumar P., Singh J., Dhanda S. (2023). Impact of Probiotics in Alleviating Type 2 Diabetes Risk in Clinical Trials: A Meta-Analysis Study. Hum. Gene.

[24] Grundy S.M., Cleeman J.I., Daniels S.R., Donato K.A., Eckel R.H., Franklin B.A., Gordon D.J., Krauss R.M., Savage P.J., Smith S.C. (2005). Diagnosis and Management of the Metabolic Syndrome: An American Heart Association/National Heart, Lung, and Blood Institute Scientific Statement. Circulation.

[25] Callahan B.J., McMurdie P.J., Rosen M.J., Han A.W., Johnson A.J.A., Holmes S.P. (2016). DADA2: High-Resolution Sample Inference from Illumina Amplicon Data. Nat. Methods.

[26] McMurdie P.J., Holmes S. (2013). Phyloseq: An R Package for Reproducible Interactive Analysis and Graphics of Microbiome Census Data. PLoS ONE.

[27] Anders S., Huber W. (2010). Differential Expression Analysis for Sequence Count Data. Genome Biol..

[28] Love M.I., Huber W., Anders S. (2014). Moderated Estimation of Fold Change and Dispersion for RNA-Seq Data with DESeq2. Genome Biol..

[29] Dixon P. (2003). VEGAN, a Package of R Functions for Community Ecology. J. Veg. Sci..

[30] Douglas G.M., Maffei V.J., Zaneveld J.R., Yurgel S.N., Brown J.R., Taylor C.M., Huttenhower C., Langille M.G.I. (2020). PICRUSt2 for Prediction of Metagenome Functions. Nat. Biotechnol..

[31] Kappel B.A., De Angelis L., Heiser M., Ballanti M., Stoehr R., Goettsch C., Mavilio M., Artati A., Paoluzi O.A., Adamski J. (2020). Cross-Omics Analysis Revealed Gut Microbiome-Related Metabolic Pathways Underlying Atherosclerosis Development after Antibiotics Treatment. Mol. Metab..

[32] Ballanti M., Antonetti L., Mavilio M., Casagrande V., Moscatelli A., Pietrucci D., Teofani A., Internò C., Cardellini M., Paoluzi O. (2024). Decreased Circulating IPA Levels Identify Subjects with Metabolic Comorbidities: A Multi-Omics Study. Pharmacol. Res..

[33] (2025). 61st EASD Annual Meeting of the European Association for the Study of Diabetes. Diabetologia.

