# Supplementary material for: Use of 16S rRNA Gut Microbiome Analysis to Identify Shared Microbial Signatures Across Metabolic Syndrome Components in Obese Subjects
Source: Int J Mol Sci. 2026 Oct 29;27(21):9701. doi: 10.3390/ijms27219701 (PMC13762712; doi:10.3390/ijms27219701)
Supplement: Supplementary file 1 [file ijms-27-09701-s001.zip › ijms-4600235-supplementary.pdf]

5. Supplementary

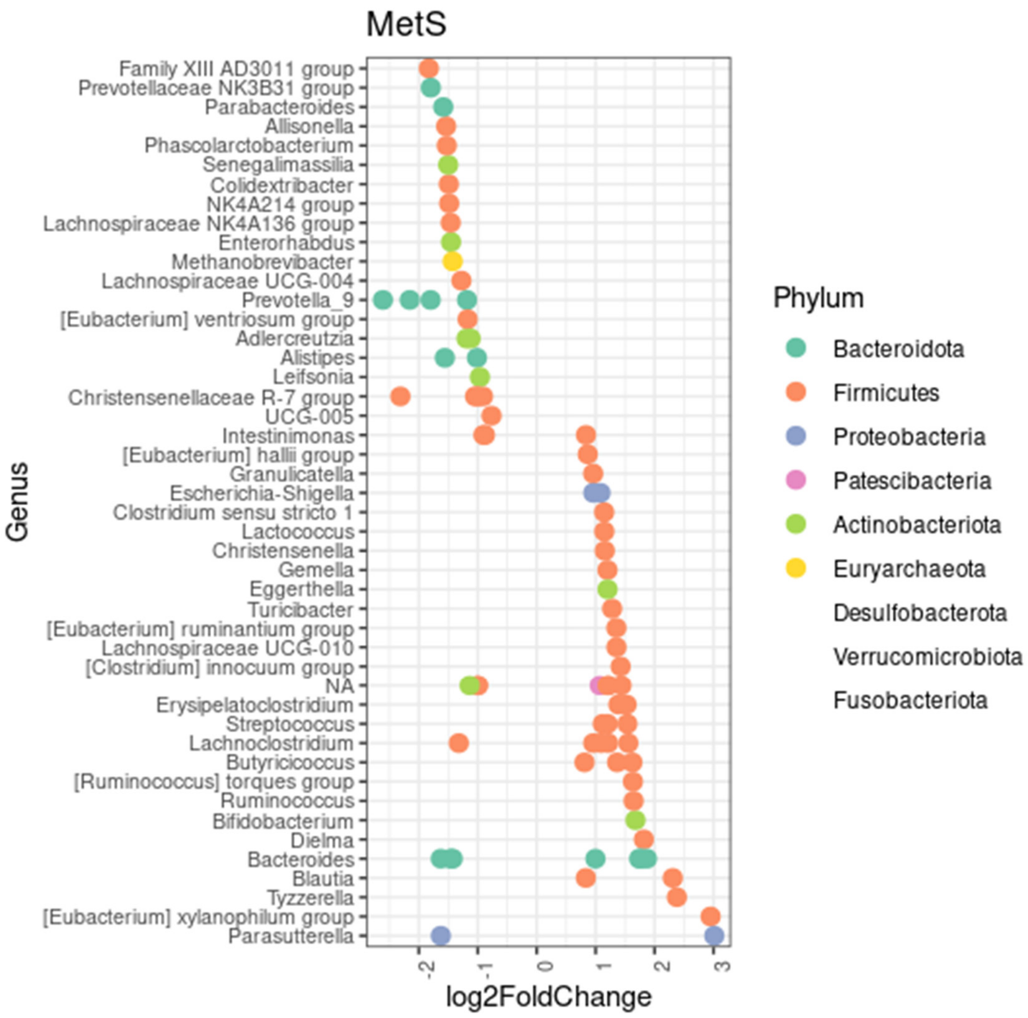

Figure S1: *Comparative analysis by DESeq2 between individuals with metabolic syndrome and controls. Only ASVs with BH-adjusted p-value < 0.05 are shown.*

Trait	N	R2	F_statistic	p_value	p_adj_BH	betadisper_p
Hyperglycemia	174	0,0092	1,603	0,012	0,02	0,1948
Triglycerides	167	0,0085	1,415	0,035	0,035	0,3573
Hypertension	161	0,0101	1,626	0,012	0,02	0,1224
HDL	167	0,0085	1,415	0,03	0,035	0,3573
MetS	176	0,0097	1,702	0,008	0,02	0,1898

Table S1: *PERMANOVA results for beta-diversity analysis across MetS traits. For each metabolic trait, samples lacking data for that trait were excluded. PERMANOVA was performed using 999 permutations with MetS trait status (absent vs present) as grouping variable (adonis2, vegan package). p_adj: Benjamini-Hochberg correction across 5 tests. betadisper p: p-value from Anderson's test of multivariate homogeneity of group dispersions; non-significant values (p > 0.05) indicate homogeneous within-group dispersion, supporting the validity of PERMANOVA results.*

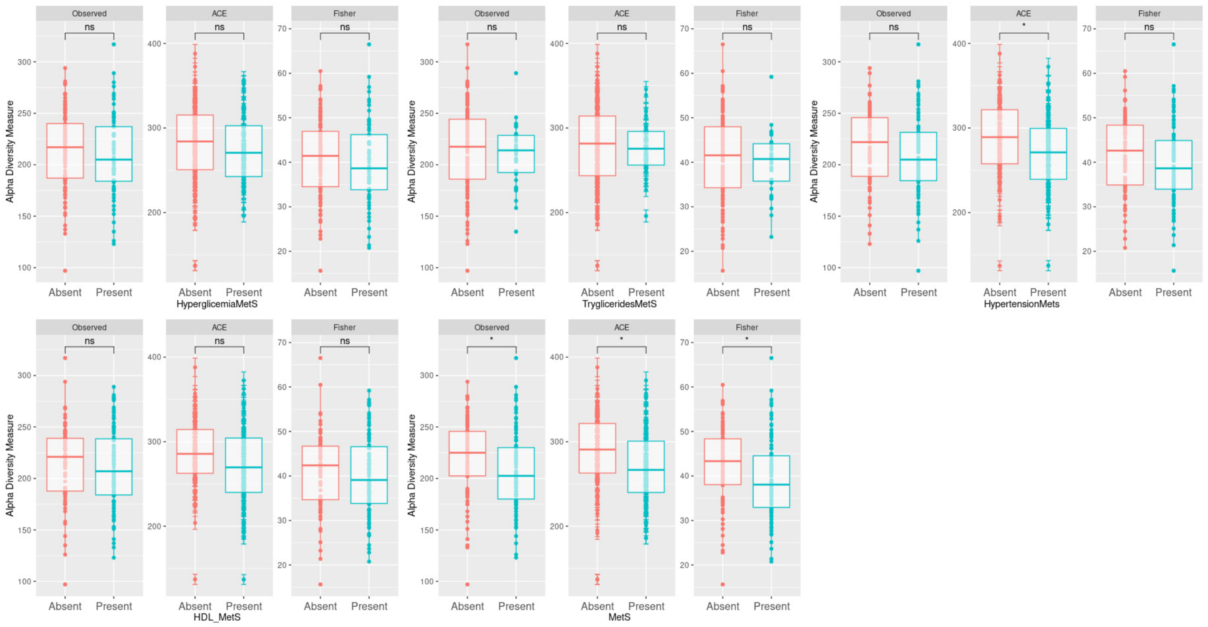

Figure S2: *Alpha-diversity analysis across metabolic syndrome traits calculated on rarefied 16S rRNA gene amplicon sequencing data. Boxplots show Observed, ACE, and Fisher indices for each MetS component (Hyperglycemia, Triglycerides, Hypertension, HDL, and MetS overall). ns indicates non-significant differences between trait-positive and trait-negative groups.*

Variable	N	Mean ± SD / %
Age (years)	37	59.1 ± 8.8
BMI (kg/m^2)	37	31.2 ± 8.9
SBP (mmHg)	37	124.7 ± 11.5
DBP (mmHg)	37	77.2 ± 8.0
Fasting plasma glucose (mg/dL)	37	99.9 ± 30.1
HbA1c (mmol/mol)	37	38.4 ± 10.1
Triglycerides (mg/dL)	37	112.5 ± 67.2
HDL cholesterol (mg/dL)	37	58.2 ± 16.9
Total cholesterol (mg/dL)	37	194.8 ± 36.6
HOMA-IR	37	3.3 ± 2.3
CRP (mg/L)	30	3.4 ± 6.3
Sex (% female)	37	48.6%
Diabetes (% yes)	37	16.2%
Hypertension (% yes)	37	54.1%
Hyperglycemia (% yes)	37	29.7%
Hypertriglyceridemia (% yes)	37	16.2%
Low - HDL Cholesterol(% yes)	37	24.3%
Abdominal obesity (% yes)	37	70.3%
Metabolic Syndrome (% yes)	37	40.5%

Supplementary Table S2: *Baseline characteristics of the external validation cohort. Continuous variables are reported as mean ± standard deviation, and categorical variables as percentages. N indicates the number of available observations. BMI: body mass index; SBP: systolic blood pressure; DBP: diastolic blood pressure; HbA1c: glycated hemoglobin; HDL: high-density lipoprotein; HOMA-IR: homeostatic model assessment of insulin resistance; CRP: C-reactive protein; MetS: metabolic syndrome.*
